# Subclavian Venous Spasm During Pacemaker Implantation: A Case Report

**DOI:** 10.7759/cureus.91482

**Published:** 2025-09-02

**Authors:** Juwayria A Ahmed, Wasifuddin Syed, Khudheeja A Ahmed, Mohammed Habeeb Ahmed

**Affiliations:** 1 Research, KAAJ Healthcare, San Jose, USA; 2 Medical School, Edward Via College of Osteopathic Medicine, Monroe, USA; 3 College of Osteopathic Medicine, Touro University California, Vallejo, USA; 4 Cardiology, KAAJ Healthcare, San Jose, USA

**Keywords:** axillary venous spasm, bradycardia, cardiac pacemaker, imaging, leadless pacemaker, pneumothorax, subclavian venous spasm

## Abstract

We present the case of an 88-year-old female who presented to the cardiologist’s clinic with symptoms of palpitations, dizziness, and fatigue. After cardiac monitoring, the patient was diagnosed with symptomatic bradycardia and required a pacemaker. During the pacemaker implantation procedure, attempts to access the subclavian vein were unsuccessful, and venography revealed severe venous spasm. The spasm did not relieve with time or nitroglycerin. Eventually, a leadless pacemaker was successfully implanted. We propose leadless pacemaker implantation as a viable management strategy for refractory venous spasm that avoids the risks of contralateral access.

## Introduction

Pacemaker lead implantation requires venous access primarily through the cephalic, subclavian, or axillary vein [1-3]. Subclavian venous access is a commonly used route with a high success rate but is also associated with a 0.6-5% risk of pneumothorax, which may increase with repeated attempts [1]. Venous spasm is a rarely reported complication during pacemaker implantation [4]. Depending on the severity, venous spasm may result in procedural delays, repeated venous access attempts with increased risk of complications, or even termination of the procedure [1,4-7]. The mechanism of venous spasm is not well understood and is hypothesized to occur due to the chemical irritation of the contrast agent or due to the vessel strain caused by multiple punctures and the insertion of the guidewire [4]. Due to the scarcity of research, there is no standard treatment for the management of venous spasm during pacemaker implantation [4]. Pre-procedural administration of intravenous nitroglycerin has been shown to lower the incidence of venous spasm during contrast-guided axillary venous puncture [4].

We found three reports in the literature of isolated venous spasm [1,6,8]. A report by Krishnappa et al. described the case of a 45-year-old woman who presented with subclavian venous spasm during pacemaker implantation [1]. Due to low systolic blood pressure, nitroglycerin was not administered, and contralateral access was not preferred as the pocket had already been opened [1]. Instead, medial subclavian vein puncture was successfully performed after venography showed retrograde flow medially in the subclavian vein [1]. Another case documented by Koza et al. reported refractory venous spasm that did not respond to nitroglycerin, which was ultimately managed by switching to a hydrophilic guidewire [6]. Finally, a Turkish pediatric cardiology center reported a case of a six-year-old child with tetralogy of Fallot and third-degree atrioventricular (AV) block who exhibited severe subclavian venous spasm upon left subclavian vein puncture during an attempt to change the battery of a previously implanted pacemaker [8]. The spasm did not respond to administration of nitroglycerine for over half an hour, and the battery was replaced via abdominal epicardial surgery instead [8]. This is the only reported pediatric case of subclavian venous spasm [8].

We found three cases in the literature of simultaneous venous spasm of both the subclavian and axillary veins [4,5,7]. A report by Vemuri et al. documented the case of a 72-year-old female presenting with both subclavian and axillary venous spasm during pacemaker implantation that did not respond to nitroglycerin [4]. The spasm was managed by contralateral access of the right axillary vein; however, this was done using anatomical landmarks and not venography, as the authors suspected the spasm to likely be a reaction to the contrast agent [4]. Another report by Oktavio et al. documented the case of subclavian and axillary venous spasm in a 55-year-old female undergoing pacemaker implantation [5]. Fortunately, the spasm responded to administration of isosorbide dinitrate, allowing the pacemaker to be successfully implanted [5]. Finally, Venet et al. reported the case of subclavian and axillary venous spasm in a 66-year-old female undergoing pacemaker implantation [7]. Unfortunately, the spasm did not respond to administration of isosorbide dinitrate, and the procedure was ultimately terminated [7].

This case adds to the literature by being among the first reports to document leadless pacemaker implantation as a successful approach to manage severe subclavian venous spasm while avoiding the usual complications.

## Case presentation

An 88-year-old female presented to the cardiologist’s office with complaints of palpitations, dizziness, and fatigue. The patient had an episode of syncope about one month before the initial office visit. She had a medical history of hypertension, chronic renal insufficiency, dyslipidemia, cardiomegaly, hypothyroidism, and arrhythmias. She also had a history of a hysterectomy done about 40 years ago and a breast biopsy.

Electrocardiography revealed sinus rhythm, left anterior fascicular block, and incomplete right bundle branch block (RBBB) with marked left axis deviation. Ambulatory cardiac monitoring showed sinus rhythm with high-grade as well as third-degree AV block. An echocardiogram showed no significant systolic dysfunction. The patient was recommended for a pacemaker implantation, but refused. Additionally, COVID-19 regulations were in effect, due to which she was unwilling to consider any procedure.

Four months later, due to worsening symptoms, the patient returned to the office. Electrocardiography showed sinus rhythm with sinus bradycardia with a heart rate of 44 beats/minute, age-indeterminate septal infarct, high lateral infarct, and RBBB. Ambulatory cardiac monitoring showed sinus rhythm, high-grade AV block, episodes of third-degree AV block, and a lowest heart rate of 30 beats/minute, with a longest pause of 2.6 seconds. As symptoms progressed, the patient agreed to undergo pacemaker implantation.

**Figure 1:**
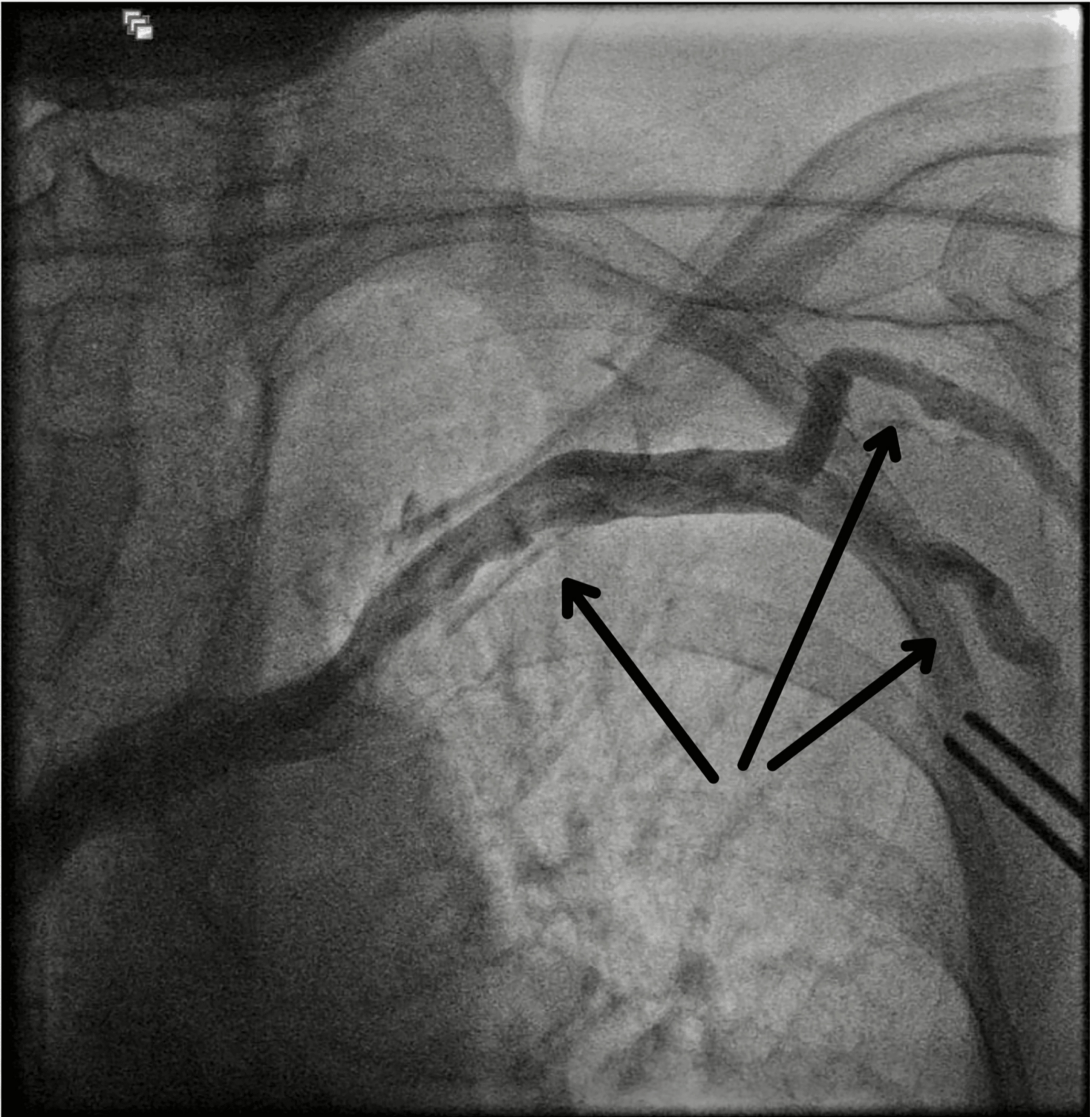
Patency of the left subclavian vein before the start of the procedure. Pre-procedural venography of the left subclavian vein provided anatomical visualization. The vein appeared to be patent and of sufficient size. The arrows are showing a patent subclavian vein and its tributary.

**Figure 2:**
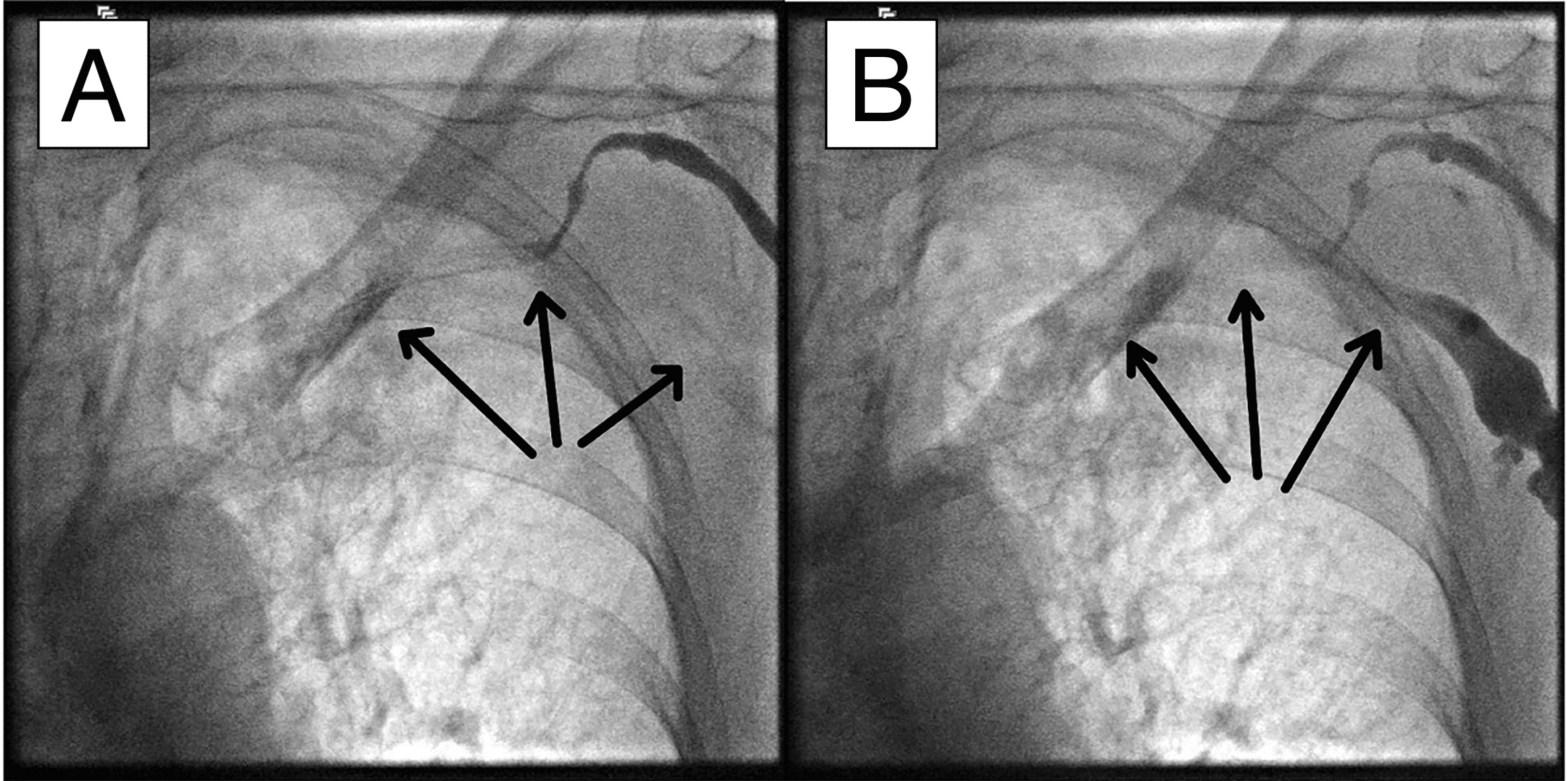
Severe left subclavian venous spasm post-access attempt. After unsuccessful attempts to access the left subclavian vein, venography was repeated and revealed complete spasm of the entire left subclavian vein. Two panels from the venography are shown. Reduced blood flow in the affected segments is indicated by arrows.

**Figure 3:**
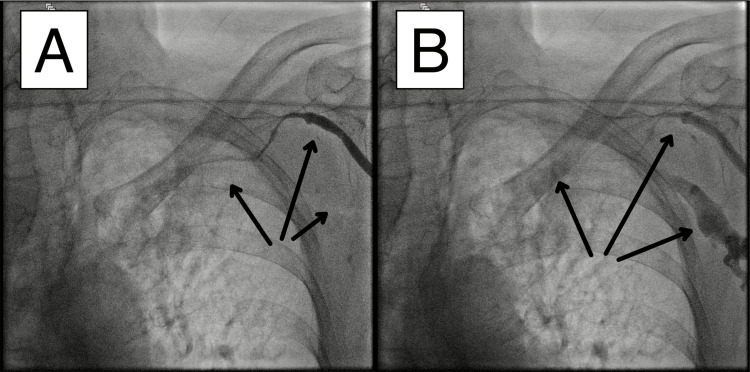
Refractory venous spasm post-administration of intravenous nitroglycerin. After administration of intravenous nitroglycerin, venography was repeated to assess the status of the spasm. Two panels from the venography are shown. The spasm had not resolved, requiring the pacemaker implantation procedure to be aborted. Reduced blood flow in the affected segments is indicated by arrows.

The patient was diagnosed with symptomatic bradycardia due to high-grade and third-degree AV block. To evaluate and manage the patient’s angina and symptomatic bradycardia, the patient was brought into the cardiac catheterization laboratory. First, left heart catheterization was performed at 8:30 am, which found luminal irregularities in the left anterior descending artery with a mid eccentric 40-50% stenosis. As there was no significant coronary artery disease, we proceeded with a permanent pacemaker implantation. The patient was prepped and draped in the usual sterile fashion. At 9:02 am, intravenous venography was performed to visualize the left subclavian vein, which appeared to be normal in size with no angiographic abnormalities visualized (Figure 1, Video 1). Local anesthesia was administered in the left infraclavicular space. Following adequate anesthesia, an incision was made, and the pocket was fashioned using sharp and blunt dissections. After appropriate hemostasis, an attempt was made to gain access to the left subclavian vein at 9:20 am. After a few attempts, the access could not be obtained, even after switching to a hydrophilic guidewire. Intravenous venography was again performed at 9:25 am to better visualize the vein, which revealed subtotal occlusion of the left subclavian vein (Figure 2, Video 2). Nitroglycerin was administered intravenously at an infusion rate of 5 µg/minute. A maximum of 100 µg was administered. This was determined based on the patient’s systolic blood pressure. Venography was again performed at 9:32 am, showing refractory spasm with subtotal occlusion (Figure 3, Video 3).

**Video 1:** Patency of the left subclavian vein before the start of the procedure.

**Video 2:** Severe left subclavian venous spasm post-access attempt.

**Video 3:** Refractory venous spasm post-administration of intravenous nitroglycerin.

**Figure 4:**
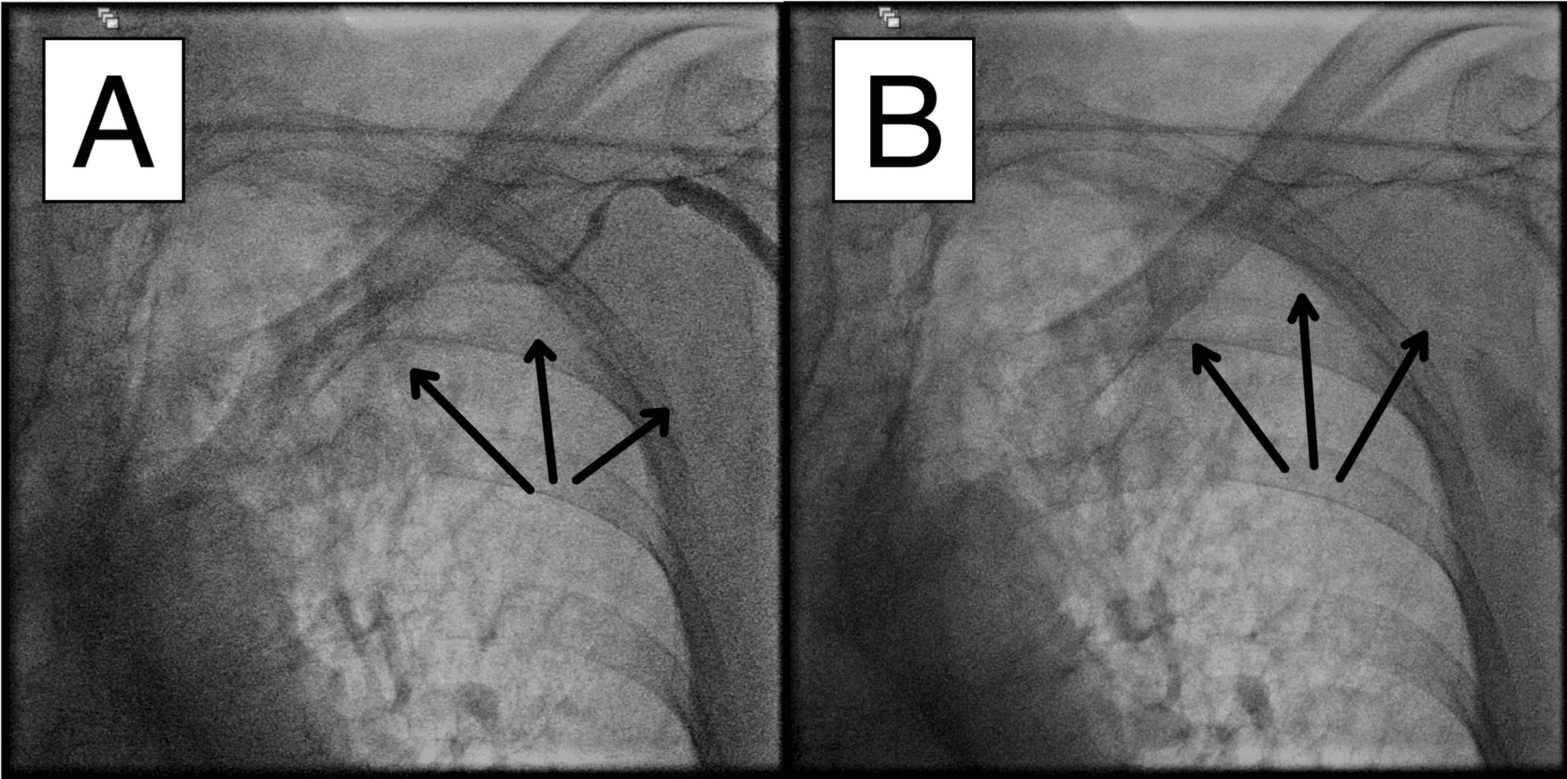
Refractory severe venous spasm unresolved almost an hour after the initial onset. After implanting the temporary pacemaker, venography was repeated to reassess vein accessibility. Two panels from the venography are shown. However, the spasm had still not resolved, eliminating the possibility to resume the initial pacemaker implantation procedure. Reduced blood flow in the affected segments is indicated by arrows.

As the patient was fluctuating between high-grade and third-degree AV block, the procedure was aborted, and a temporary transvenous pacemaker was implanted via a right groin femoral approach. Intravenous venography was again performed at 9:53 am. The vein still appeared to be in spasm, showing persistent subtotal occlusion (Figure 4, Video 4). There were no obvious signs of pneumothorax fluoroscopically, with clearly visible lung markings and hemodynamic stability. The patient was transported to the intensive care unit (ICU). The pocket was not closed, as it was hoped that the subclavian venous spasm would resolve shortly.

**Video 4:** Refractory severe venous spasm unresolved almost an hour after the initial onset.

At the ICU, a chest X-ray performed at 10:30 am showed moderate left pneumothorax, approximately 15% of the thoracic cavity. It showed likely left upper lobe atelectasis; however, infiltrate could not be ruled out. The interventionist then proceeded with closure of the pacemaker pocket, after which the patient remained hemodynamically stable in the ICU for 72 hours. Three days later, the patient was brought back to the cardiac catheterization laboratory and underwent successful leadless pacemaker implantation via a right groin femoral approach.

**Figure 5:**
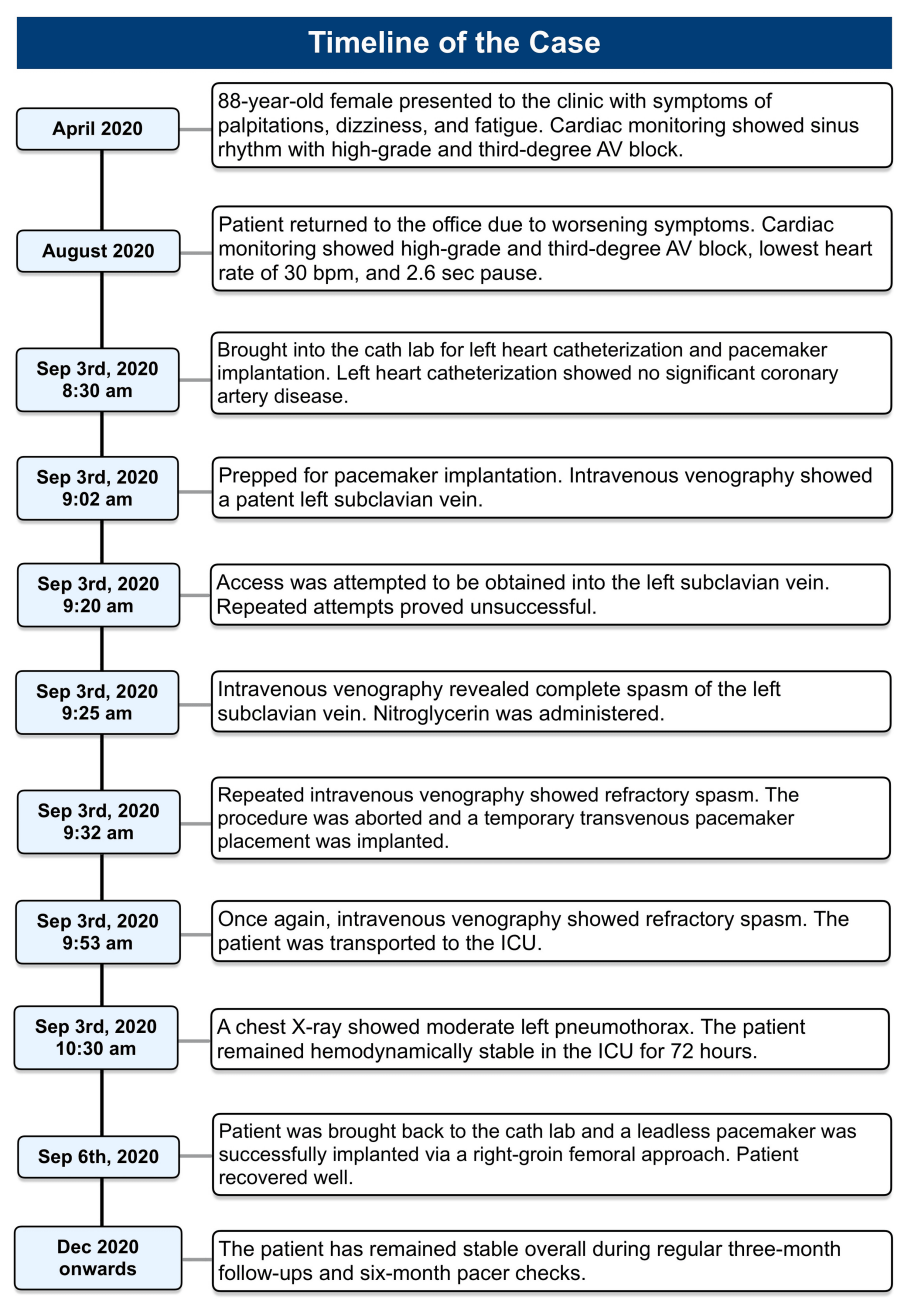
Timeline of the case.

The patient recovered well post-pacemaker implantation, reporting overall improvement and no post-discharge symptoms. She has been attending regular three-month follow-ups and six-month pacer checks for the past three to four years, remaining stable overall. A visual summary depicting the timeline of the case is shown in Figure 5. Informed patient consent was obtained from the patient for publication of this report and any accompanying images.

## Discussion

Subclavian venous spasm is a rare but significant complication during pacemaker implantation, particularly when it is severe and unresponsive to vasodilators or time. While also rare, axillary venous spasm occurs during pacemaker implantation with a higher incidence than subclavian venous spasm. One study found 12 cases of axillary venous spasm, managed by switching to subclavian vein puncture, and no cases of subclavian venous spasm from 403 cardiac implantable electronic device implantation procedures. The authors hypothesized that the patency of the lumen of the subclavian vein is supported by the two adjacent bony structures [9]. We found six reports of axillary venous spasm during pacemaker implantation, some of which resolved spontaneously over time or 10 minutes after the administration of intravenous nitroglycerin, while others required a change of access site or contralateral venous access [1-4,10,11]. However, these solutions are not without risk as multiple punctures increase the risk of complications, and contralateral access may result in a similar venous spasm, which has no clear solution, except for possibly a cephalic vein cut-down technique described in a single case report [3,4].

We present the case of an elderly patient exhibiting subclavian venous spasm during pacemaker implantation. In our case, the venous spasm was severe, refractory to nitroglycerin, and persistent even after a significant time had passed. Hydrophilic guidewire use was pursued initially, but was unsuccessful due to the intensity of the spasm. Due to the presence of an evolving pneumothorax as well as the patient’s medical history of multiple comorbidities, including chronic renal insufficiency and cardiomegaly, procedural risk was heightened. These factors made contralateral venous access less desirable, as a similar complication could have arisen. Furthermore, leadless pacemaker implantation avoided any further venous access attempts within the affected area, thereby preventing increased risk of pneumothorax.

The cephalic vein cutdown method was not considered, as it would have offered only distal access and no advantage, given that the hydrophilic guidewire could not access the subclavian vein due to persistent spasm. Therefore, under these circumstances, the leadless pacemaker provided a minimally invasive, efficient management strategy, bypassing the compromised venous anatomy entirely while avoiding further procedural trauma and reducing the risk of bilateral complications.

Based on our case, we propose the implantation of a leadless pacemaker as a potential option that avoids risks associated with contralateral access and multiple punctures when faced with severe refractory subclavian venous spasm during pacemaker implantation [1,3,4]. However, leadless pacemaker implantation is also associated with several risks, ranging from lead fracture, dislodgement, infection, pericardial effusion, cardiac tamponade, and thrombosis [12]. These risks of leadless pacemaker implantation should be taken into consideration. However, leadless pacemakers have a better overall safety profile than conventional pacemakers, with lower pocket site and lead-related infections than conventional pacemakers [12].

## Conclusions

Subclavian venous spasm is a rarely reported complication of pacemaker implantation that can prevent venous access and has no established management strategy. While approaches such as nitroglycerin, hydrophilic guidewires, or contralateral access have been attempted, each carries risks and limitations. In our case, the spasm was severe, refractory to nitroglycerin, and complicated by pneumothorax risk. Implantation of a leadless pacemaker via femoral access provided a safe and effective alternative. This strategy may be considered in similar high-risk or refractory cases, although further studies are needed to establish its role in clinical practice.
